# Resolvin D2 Induces Resolution of Periapical Inflammation and Promotes Healing of Periapical Lesions in Rat Periapical Periodontitis

**DOI:** 10.3389/fimmu.2019.00307

**Published:** 2019-02-26

**Authors:** Yasir Dilshad Siddiqui, Kazuhiro Omori, Takashi Ito, Keisuke Yamashiro, Shin Nakamura, Kentaro Okamoto, Mitsuaki Ono, Tadashi Yamamoto, Thomas E. Van Dyke, Shogo Takashiba

**Affiliations:** ^1^Department of Pathophysiology-Periodontal Science, Okayama University Graduate School of Medicine, Dentistry and Pharmaceutical Sciences, Okayama, Japan; ^2^Department of Periodontics and Endodontics, Okayama University Hospital, Okayama, Japan; ^3^Center for Innovative Clinical Medicine, Okayama University Hospital, Okayama, Japan; ^4^Department of Molecular Biology and Biochemistry, Okayama University Graduate School of Medicine, Dentistry and Pharmaceutical Sciences, Okayama, Japan; ^5^Center for Clinical and Translational Research, The Forsyth Institute, Cambridge, MA, United States

**Keywords:** resolvin D2, resolution of periapical inflammation, periapical periodontitis, periapical lesion, DMP1, calcification

## Abstract

Periapical periodontitis results from pulpal infection leading to pulpal necrosis and resorption of periapical bone. The current treatment is root canal therapy, which attempts to eliminate infection and necrotic tissue. But, in some cases periapical inflammation doesn't resolve even after treatment. Resolvins belongs to a large family of specialized pro-resolving lipid mediators that actively resolves inflammation signaling via specific receptors. Resolvin D2 (RvD2), a metabolite of docosahexaenoic acid (DHA), was tested as an intracanal medicament in rats *in vivo*. Mechanism was evaluated in rat primary dental pulp cells (DPCs) *in vitro*. The results demonstrate that RvD2 reduces inflammatory cell infiltrate, periapical lesion size, and fosters pulp like tissue regeneration and healing of periapical lesion. RvD2 enhanced expression of its receptor, GPR18, dentin matrix acidic phosphoprotein 1 (DMP1) and mineralization *in vivo* and *in vitro*. Moreover, RvD2 induces phosphorylation of Stat3 transcription factor in dental pulp cells. We conclude that intracanal treatment with RvD2 resolves inflammation and promoting calcification around root apex and healing of periapical bone lesions. The data suggest that RvD2 induces active resolution of inflammation with pulp-like tissue regeneration after root canal infection and thus maybe suitable for treating periapical lesions.

## Introduction

Periapical periodontitis is an inflammatory disease that occurs around the tooth root apex. It is caused by infection of the dental pulp tissue subsequent to carious lesions and leads to resorption of root dentin and bone (1). It is believed that presence of microorganisms in the root canal system are associated with the development and progression of periapical periodontitis (2, 3). Conventional root canal treatment goals to eliminate bacteria from the root canal system as completely as possible by physically removing the pulp soft tissue and irrigating the root canal system with potent antiseptic solutions (e.g., sodium hypochlorite), as well as mechanically removing infected dentin in the root canal (4). The disinfected root canals are then filled with an inert material, typically *gutta percha*, to obturate the endodontic space to prevent bacterial recolonization. The overall success rates for primary endodontic, secondary endodontics, and surgical treatment reported by Elimam et al. were 86.02, 78.2, and 63.4%, respectively, based on criteria of the retention of a functional tooth over a 4 years period. (5). Most failures occur because of ineffective microbial clearance, which leads to continued periapical inflammation (6, 7). Root canal therapy also desiccates the tooth leading to the potential for subsequent fracture. Considering the course which pulpal inflammation initiates tissue destruction, it is obvious that an important step in supporting the regeneration of pulp-like tissue is the attenuation of inflammation.

Regeneration of pulp tissue after infection is limited, and attempts have repeatedly failed, because current methods cannot control inflammation and eliminate the bacterial infection (8). The primary goal in regenerative procedure is to eliminate clinical symptoms and resolve apical periodontitis as defined by the American Association of Endodontists in *Clinical Considerations for a Regenerative Procedure* (9). Thickening of canal walls and constant root maturation characterized by continued odontoblast activity, which produces calcified tissues, is the secondary goal. Currently, non-surgical root canal therapy replaces infected vital and necrotic tissue with biocompatible foreign materials in disinfected root canals. The goal of regenerative therapy is to fill formerly infected canals with the host's own vital tissue (9). Earlier, it was believed that successful regeneration cannot be achieved once tooth has become infected. However, recent studies suggest that regenerative endodontics may in fact be possible in teeth with pulpal necrosis and periapical pathology. Maintaining patency of the root apex opening is thought to be a critical component for regeneration as multiple studies in experimental animal models have revealed the regeneration of dental pulp-like tissue after evoked bleeding by instrumentation (10, 11).

Dental pulp cells (DPCs) are progenitor cells with the ability for self-renewal and multilineage differentiation. In response to trauma or injury, DPCs differentiate into odontoblast-like cells and initiate dentin mineralization by expressing extracellular acidic proteins that participate in dentin repair and mineralization (12). Dentin matrix protein-1 (DMP1) plays a key role in odontoblast differentiation, formation of the dentin tubular system, and mineralization. DMP1 is expressed by both pulp progenitor cells and odontoblasts and its deletion leads to defects in odontogenesis and mineralization (13). It has been suggested that DPCs can be transplanted or expanded in a sterile root canal to differentiate and induce mineralization and promote periapical healing (12, 14). The clinical limitation to this approach is the difficulty in controlling infection and inflammation.

Resolvins belongs to a family of lipid mediators biosynthesized from omega-3 polyunsaturated fatty acids (eicosapentaenoic acid, EPA and docosahexaenoic acid, DHA) that promote the resolution phase of inflammation. In periodontitis and other infectious/inflammatory diseases, resolvins promote clearance of bacteria, and tissue regeneration (15, 16). The lipid mediator resolvin D2 (RvD2) promotes bacterial clearance and improves animal survival in a cecal ligation and puncture model of sepsis (16). RvD2 is defensive against *P. gingivalis* provoked periodontal bone loss and has been shown to regulate the RANKL/OPG ratio (17). RvD2 enhances post-ischemic limb revascularization during ischemia by promoting arteriogenesis (18), controlling bacterial sepsis and resolving inflammation by promoting polymorphonuclear neutrophil (PMN) apoptosis, and enhancing macrophage efferocytosis (16, 19). RvD2 is also known to reduce postoperative pain by inhibiting transient receptor potential channels in sensory neurons (20). Considering the demonstrated regeneration of periodontal tissues with resolvin treatment and the demonstrated actions of RvD2 in a variety of infectious / inflammatory disease systems, it is reasonable to expect that the active proresolving actions of RvD2 and its demonstrated enhancement of bacterial clearance will be beneficial in healing of periapical lesions. We examined whether RvD2 can be used as an intracanal medication to promote periapical healing and investigated potential mechanism of action.

## Materials and Methods

### Animals

Eighteen 10-week old male Wistar rats (CLEA Japan, Inc., Tokyo, Japan) were maintained in the animal facility of Department of Animal Resources, Advanced Science Research Center, Okayama University with a 12-h light/12-h dark cycle. Food and water were provided *ad libitum*.

### Induction of Periapical Periodontitis

**Figure 1:**
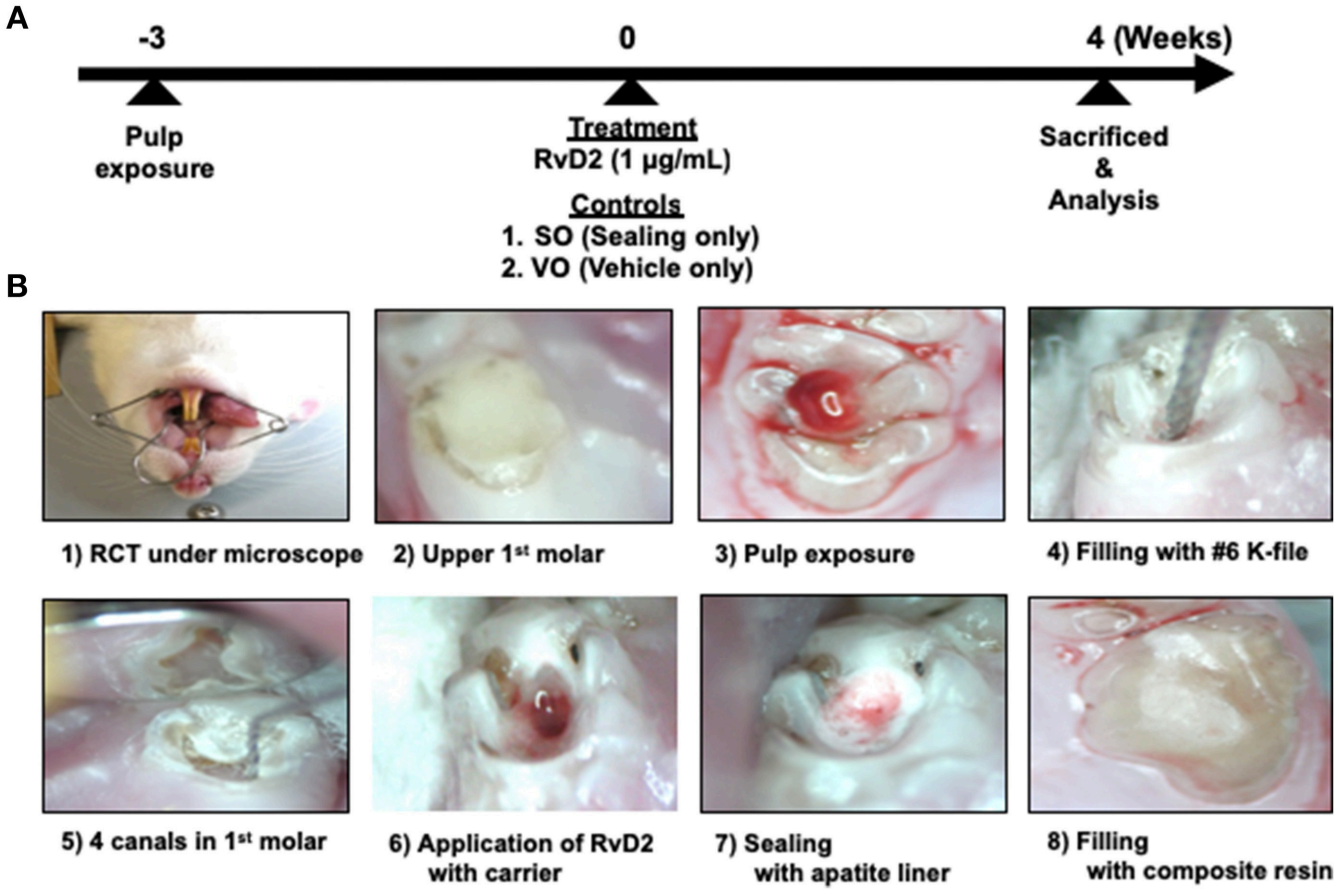
Experimental protocol of rat periapical periodontitis model. **(A)** Root canal treatment was performed 3 weeks after pulp exposure and rats were sacrificed and evaluated 4 weeks after treatment. **(B)** Cavity preparations of first molar, root canal treatment, and final restoration under microscope guidance.

The experimental design is shown in Figure 1. Rats were administered general anesthesia by intraperitoneal injection of sodium pentobarbital (50 mg/kg IP). After 15–20 min to ensure complete anesthesia, all surgical procedures were performed under a microscope (Nikon Smz-645, Tokyo, Japan). Periapical lesions were induced by exposing the pulp of the mandibular right and left first molars using a #1/2 8ISO 006 round bur (Dentsply Maillefer, Ballaigues, Switzerland) in an electric handpiece (VIVAMATE G5; NSK, Tochigi, Japan). The exposed pulps were left open to the oral environment for 3 weeks to ensure bacterial contamination. Opposing maxillary first molars in contact with the experimental teeth were removed at the same time as pulp exposure of the mandibular first molars to prevent tooth fracture (1).

After 3 weeks, the right and left mandibular first molars were endodontically treated by cleaning the test tooth with 70% ethanol. A #1/2 round bur was used to open the pulp chamber and remove the necrotic coronal pulp, and a micro-excavator (OK Micro-exca; Seto, Ibaraki, Japan) was used to remove the infected tooth substance from the pulpal floor and the orifice of the root canals. K-files #6–20 (Dentsply Maillefer) were placed up to 2.5 mm in depth into the mesial and distal root canals to mechanically debride the root canals, followed by irrigation with 0.5 mL of 2.5% sodium hypochlorite (Neo Dental Chemical Products, Tokyo, Japan) using 30-gauge needles (NaviTip, Ultradent Products, Tokyo, Japan) at a flow rate of 1 mL/min (1). In order to facilitate bleeding from periapical lesion into the canals, we breached the apical foramen using # 20 K file. Treated canals were dried using sterilized paper points (VDW, Munich, Germany) and filled with 20 μL RvD2 (1 μg/mL, Cayman Chemical, Ann Arbor, MI, USA) delivered with a mixture of propylene glycol (Nacalai Tesque) and Macrogol (Meiji Yakuhin, Toyama, Japan) as a carrier with 30-gauge needles (21, 22). K file #15 was used in a clockwise motion to facilitate RvD2 delivery inside the canal and beyond the apex, until RvD2 changed from colorless to reddish color due to blood. This was followed by drying of the coronal chamber using sterile cotton. Untreated mandibular molars, sealed only (SO group; same treatment without any administration) and with vehicle only (VO group; a mixture of propylene glycol and Macrogol) served as controls. Finally, pulp chambers were lined with apatite liner (Dentsply-Sirona, Tokyo, Japan) followed by filling with flowable composite resin (MI FLOW, GC, Tokyo, Japan). In this study. 13 rats were used for the test with control group (Group #1); RvD2 (right side) and SO (left side) sides for each rat. On the other hand, 3 rats were used for control only group (VO group; Group #2). Two rats were used for the baseline group; Group #3. For *in vivo* imaging analysis, 3 rats used were from Group #1. For micro-CT analysis, 4 rats were used from Group #1. For histology, Gram staining, and immunohistochemistry, 5 rats were used from group #1, 3 from Group #2, and 2 from Group #3. In addition, for q-PCR analysis for bacteria (Figure S3), 1 rat was used from Group #1.

### *In vivo* Imaging

After 4 weeks of treatment, *in vivo* imaging was performed to measure myeloperoxidase (MPO) activity of activated phagocytes. Dose of XenoLight RediJect inflammation probe (PerkinElmer, Waltham, MA) was calculated and administered intraperitoneally at 150 μL/30-g weight, and sacrificed immediately. To eliminate errors in measurement due to positional effects of the specimen, the dissected mandibles were trimmed to the same size and thickness. After verifying that the wavelengths from specimens positioned on a plate and from the emission filters of the device were almost the same across all samples, luminescent images were taken using a charge-coupled-device (CCD) camera within 20 min of injection. Luminescence intensity was measured using IVIS Spectrum (PerkinElmer), and a circular region of interest (ROI) was defined as a region which exhibited more than 50% of maximum luminescence in the inflammatory site of each rat. The total flux (measured in photons per second) in the ROI were quantified using Living Image Software V4.4 (PerkinElmer) according to the manufacturer's instructions (23).

### Micro-CT Analysis

Periapical lesions were scanned with a μCT scanner (SkyScan1174v2; Bruker-μCT, Billerica, MA, USA) 4 weeks after treatment. After scanning, the image data were reconstructed using the Nrecon system (Nrecon Bruker-μCT). For visualization, samples were digitally reconstructed so that a two-dimensional slice showing a patent mesial and distal canal in the first molar could be obtained. Periapical lesion sizes in the cross sectional area from the apical third of the canals were measured using ImageJ software (NIH, Bethesda, MD, USA), and values in square millimeters were compared between the RvD2 and control groups (24). To obtain the precise periapical lesion size, the periodontal ligament space around the apical third of the mesial and distal root canals in healthy teeth were measured, and their values were subtracted from the periapical lesion size values of the RvD2 and control groups.

### Histology

Rats were sacrificed 4 weeks after treatment. Mandibular samples containing the first molars were dissected, fixed in 4% paraformaldehyde, and decalcified in 10% formic acid for 10 days at room temperature. After preparation of 4-μm serial sections, some sections were stained with hematoxylin and eosin (HE) to observe cellular composition, while others were stained using a modified Brown and Brenn method (25), to observe microorganisms (Gram-positive or Gram-negative) under a DP70 light microscope (Olympus, Tokyo, Japan).

### Immunohistochemistry

Immunohistochemistry was performed using a streptavidin-biotin complex method. Rabbit anti-GPR18 polyclonal antibody (Abcam, Cambridge, UK) at a 1:100 dilution and rabbit anti-DMP1 polyclonal antibody (Takara Bio, Shiga, Japan) at 1:200 dilutions were used as primary antibodies as previously described (26). Secondary antibodies were goat anti-rabbit conjugated with biotin antibody (Vectastain ABC kit; Vector Laboratories, Burlingame, CA, USA) at 1:200 dilution. Immunoreactivity was visualized using the DAB Substrate Kit (Vector Laboratories) and counterstained with Mayer's hematoxylin solution. Negative control staining was performed in parallel by incubating the sections with phosphate-buffered saline (PBS) rather than with primary antibody.

### DPC Isolation and Culture

After sacrifice, the incisors from animals were carefully separated from the jawbones. Dental pulps were gently isolated using a sterile dental explorer and the apical third of the tooth was cut off to obtain the apical epithelial buds as previously described (27). The remaining pulp tissue was minced into small pieces and treated with solution containing 3 mg/mL collagenase type 1 (Sigma-Aldrich, St. Louis, MO, USA) and 4 mg/mL dispase (Sigma-Aldrich) for 60 min at 37°C. The single-cell suspension was cultured in alpha minimum essential medium (α-MEM; Gibco, Life Technologies, Grand Island, NY, USA) supplemented with 20% fetal bovine serum (Hyclone, Logan, UT, USA), 1% 100 U/mL penicillin, 100 μg/mL streptomycin, 0.25 μg/mL amphotericin B, and 1% L-glutamine at 37°C in 5% CO_2_. The cells were routinely observed under a TS100-F phase-contrast inverted microscope (Nikon, Tokyo, Japan). Cells at passage four were used in subsequent experiments. To treat DPCs with RvD2, osteogenic medium containing 200 μM ascorbic acid, 10 mM, ß-glycerophosphate, and 100 nM dexamethasone was prepared.

### Alizarin Red S Staining

Cells were cultured on 48-well plates, and the five different groups (0, 1, 10, 100, and 200 nM) were established. Media with and without RvD2 were replaced twice per week with freshly-prepared osteogenic media. On day 21, cells were fixed with phosphate-buffered formalin and then stained with 250 μL alizarin red, ARD-A1 (ARD-SET, PG Research, Tokyo, Japan) for 30 min. After washing the wells with pure water, the plates were photographed. After staining, quantitative analysis of mineralization was carried out using 250 μL ARD-E1 (ARD-SET, PG Research, Tokyo, Japan) and plates were stirred for 10 min to elute the dye, and then 100 μL of solution was transferred into 96-well plates and absorbance measurement was recorded at 450 nm using microplate reader (iMark™ Microplate Absorbance Reader, Bio-Rad, Hercules, CA, USA) (28).

### Real-Time Reverse Transcription Polymerase Chain Reaction (Real-Time RT-PCR)

DPCs were cultured in 12-well plates until 80% confluence, treated with 0–100 nM doses of RvD2, and incubated at 37°C for 7 and 14 days. Every 72 h, the media containing RvD2 doses were changed. For real-time RT-PCR, the cells were lysed to extract total RNA using the RNeasy Mini Kit (Qiagen GmbH, Hilden, Germany). Concentrations of mRNA were measured spectrophotometrically using a NanoDrop 2000 spectrophotometer (Thermo Fisher Scientific, Waltham, MA, USA). One microgram of each RNA sample was subjected to RT using the SuperScript IV VILO cDNA synthesis kit (Invitrogen, Carlsbad, CA, USA). Quantitative RT-PCR was performed using an ABI 7300 system (Applied Biosystems, Foster City, CA) under conditions of 95°C for 10 min followed by 40 cycles at 95°C for 15 s and 60°C for 1 min in 96-well plates in a final volume of 20 μL containing SYBR green PCR master mix (Applied Biosystems) (23). The primers used for detection are listed in Table 1. Measured mRNA levels were normalized to the mRNA copies of β-actin. We performed these experiments using three different cell samples and quantification of mRNA was confirmed using the same cell sample in triplicate.

**Table 1:** Primers used in the study.

**GPR18**	
Forward	AAATGATCACCCTGAACAATCAAGA
Reverse	ATTCATAACATTTCACTGTTTATATTGCTTAG
**DMP1**	
Forward	ACCTTTGGAGACGAAGACAATGGC
Reverse	ACACCACACAGTCCAGTGAAGACA
**BETA ACTIN**	
Forward	TGTTGCCCTAGACTTCGAGCA
Reverse	GGACCCAGGAAGGAAGGCT

### Western Blot Analysis

Western blotting was performed as described previously (29). DPCs were treated with or without RvD2 (1–100 nM) for 14 days, washed with cold PBS twice, lysed using cell lysis buffer containing 50 mM sodium chloride, 10 mM, Tris-HCl (pH 7.2), 1% sodium dodecyl sulfate (SDS), 1% Nonidet P-40, and 5 mM sodium ethylenediamine tetraacetate, and collected in a 1-mL centrifuge tube. After centrifugation of the cell lysate, supernatants containing total protein were transferred to new tubes, and protein concentration was determined with the BCA protein assay (Thermo Fisher Scientific). Thirty micrograms of each sample were subjected to 12% SDS-PAGE and transferred to a polyvinylidene fluoride membrane (Bio-Rad). Membranes were incubated overnight at 4°C with a polyclonal rabbit anti-DMP1 antibody (Takara Bio) at a dilution of 1:2,000. Subsequently, the membrane was incubated with anti-rabbit IgG polyclonal antibody (GE Healthcare Life Sciences, Little Chalfont, UK) at a dilution of 1:2,000, and then washed with PBS containing Tween (PBST) buffer to remove unbound antibody. The membrane was developed with enhanced chemiluminescence detection reagents (SuperSignal West Pico; Thermo Fisher Scientific). Anti-GAPDH antibody (Cell Signaling Technology, Danvers, MA, USA) was used as an internal control at a dilution of 1:1,000). Each experiment was performed in triplicate and data values were normalized to the corresponding GAPDH values. Densitometric analysis was performed using ImageJ software.

To determine the expression levels of pSTAT3 protein, only α-MEM containing 100 nM RvD2 was used. Cells were treated with α-MEM with/without 100 nM RvD2 and incubated for 0, 1, 5, 15, and 30 min at 37°C in 5% CO_2_, followed by washing with cold PBS twice and lysis using cell lysis buffer. The lysate was added to 500-μL tubes that were transferred to ice to stop the reaction. Protein concentration was determined with the BCA protein assay and 10 μg of each sample was subjected to 12% SDS-PAGE and transferred onto a polyvinylidene fluoride membrane, followed by overnight incubation at 4°C with a polyclonal rabbit anti-pSTAT3 (Cell Signaling Technology) at a dilution of 1:1,000. Subsequently, the membrane was incubated with anti-rabbit IgG polyclonal antibody (GE Healthcare Life Science) at a dilution of 1:2,000, and then washed with PBST buffer to remove unbound antibody. Anti-STAT3 antibody (Cell Signaling Technology) was used as an internal control at a dilution of 1:1,000. Each experiment was performed in triplicate and data values were normalized to the corresponding total STAT3 values. Densitometric analysis was performed using ImageJ software.

### Statistical Analyses

For *in vivo* imaging and micro-CT analysis, Mann-Whitney test was used to determine statistically significant changes in the level of periapical inflammation and periapical lesion area between the treatment and control groups. For Alizarin red staining for mineralization, qPCR and Western blotting analysis for DMP1 mRNA and protein expression, one-way ANOVA and *post hoc* Tukey-Kramer test was used. For pSTAT3 analysis, Mann-Whitney test was used to determine statistically significant difference between RvD2 and control groups. In all cases, *P* < 0.05 was considered statistically significant (^*^).

## Results

### RvD2 Reduced MPO Activity in Periapical Periodontitis

**Figure 2:**
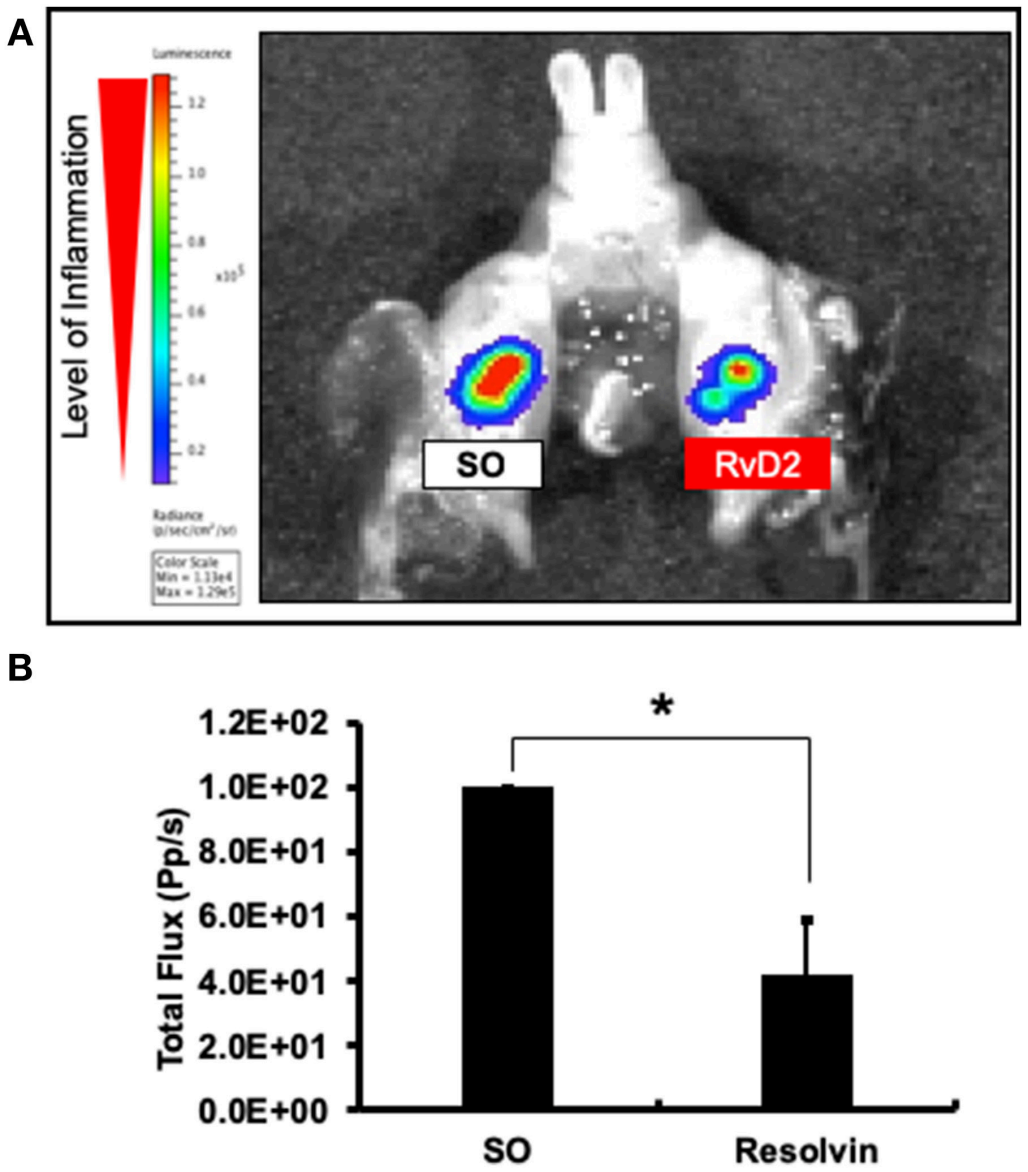
Molecular imaging analysis examining the effects of RvD2 on periapical inflammation. Images of the signal intensity of MPO activity around RvD2 treated and non-treated tooth are shown **(A)**. Results of comparisons of the levels of signal intensity with respect to total flux are shown **(B)**. Data represent the means of three independent rats (SO on left side and RvD2 on right side for each rat), with error bars indicating standard deviations. *P < 0.05 indicates significant differences compared to the control group, Mann–Whitney test.

Molecular imaging analysis was performed to measure myeloperoxidase (MPO) activity to examine the anti-inflammatory effects of the RvD2. Data revealed that level of periapical inflammation was significantly reduced in RvD2-treated molars. In the SO group, periapical inflammation was higher (Figure 2A). The average level of total flux was in RvD2 treated teeth was 42.34% ± 16.78 and for control samples set value in percentage was 100% ± 0 (^*^P < 0.05, Figure 2B).

### RvD2 Reduced Periapical Lesion Size

**Figure 3:**
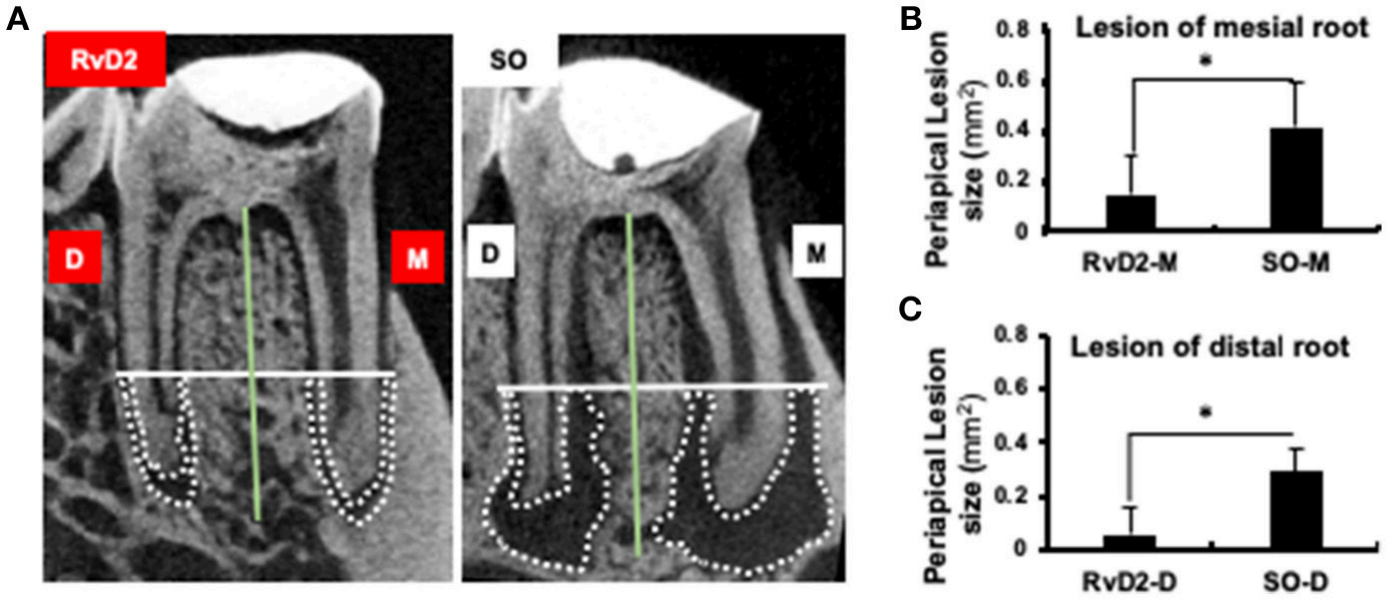
Micro-CT analysis of periapical lesions after root canal treatment in rats. **(A)** Representative image of teeth in the treatment group and SO group. The x-axis (white line) passes through the apical third of the mesial and distal root canal, denoted as the coronal limit of periapical lesion. The y-axis (green line) passes through the center of the mesial and distal root canals of the mandibular first molars. **(B,C)** Comparison of changes in the size of periapical lesions in mesial and distal canals (*P < 0.05 indicates significant differences compared to the SO group, Mann–Whitney test). Data represent the means of four independent rats (SO on left side and RvD2 on right side for each rat), with error bars indicating standard deviations. The volume of the periapical lesions of the mesial and distal roots for the treatment group were significantly lower than that of the control group after 4 weeks following pulp exposure.

Infected root canals were cleaned and sealed after administration of RvD2. Micro-computed tomography (μCT) analysis was used to quantify the area of mineralized tissue and showed that the periapical lesion size was greatly reduced, and root canal apices were calcified in RvD2-treated molars. In the SO group, root apices were open with large periapical lesions (Figure 3A). The mean periapical lesion size in RvD2 treated teeth (apex of mesial canal) was 0.149±0.146 mm^2^, and non-treated teeth was 0.391±0.119 mm^2^. The mean periapical lesion size in RvD2 treated distal canals was 0.059 ± 0.103 mm^2^, and non-treated distal canals was measured 0.292 ± 0.082 mm^2^ (^*^P < 0.05, Figures 3B,C).

### Histology

**Figure 4:**
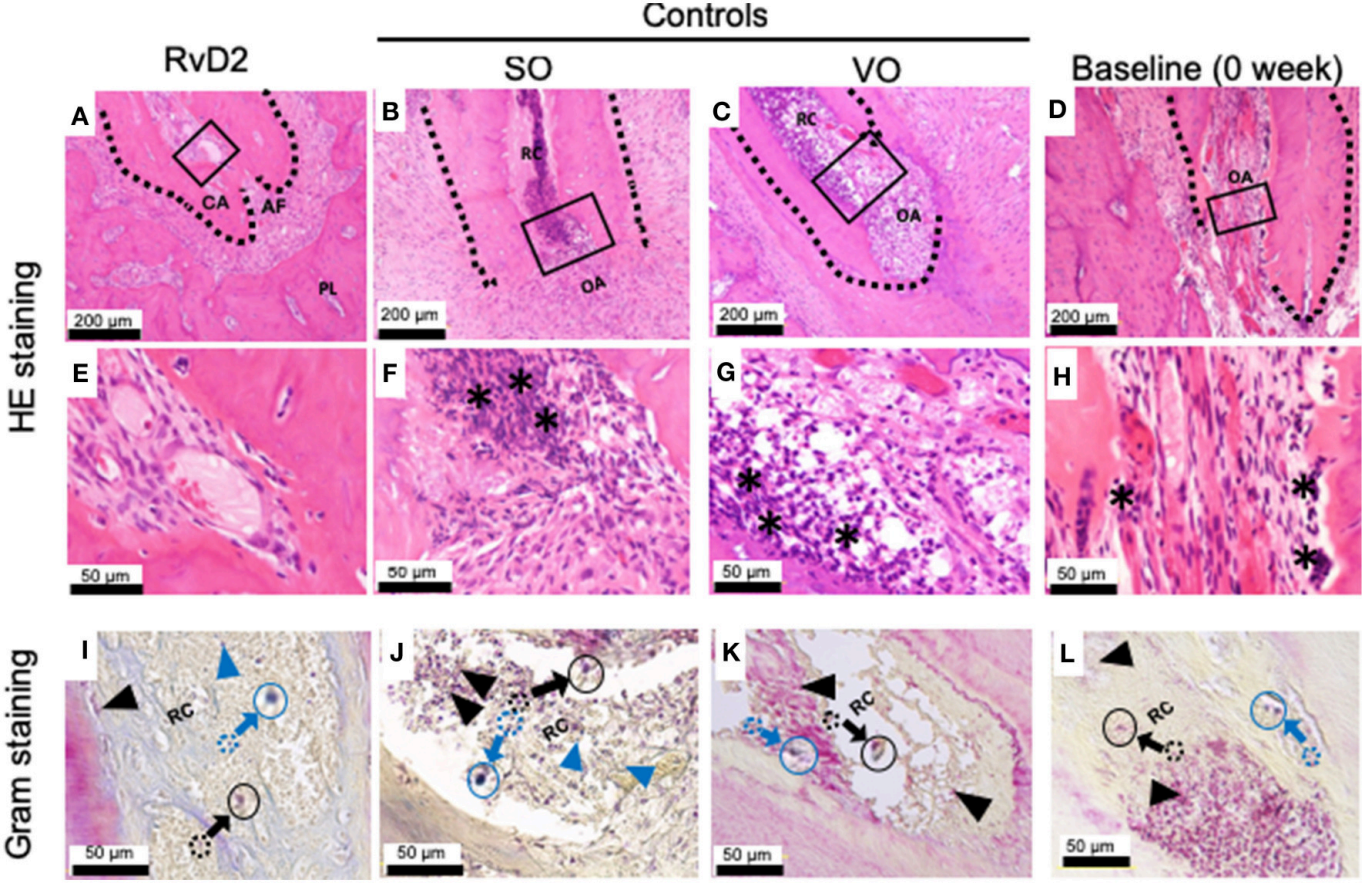
Histological analysis of periapical lesions after root canal treatment in rats. **(A)** Periapical area of treatment group stained with HE. **(B,C)** Periapical area of control groups stained with HE. **(D)** Periapical area of baseline group stained with HE. **(E,F,G,H)**. High magnification views of the solid inset in **(A–D)**, respectively. **(I–L)**. High magnification views of the solid insets in **(A–D)**, stained with a modified Brown and Brenn method. Images are representative for 5 experiments from Group #1 (RvD2 and SO); 3 experiments from Group #2 (VO), and 2 experiments Group #3 (baseline). RC, root canal; CA, closed apex; OA, open apex; AF, apical foramen. The asterisk denotes inflammatory cells and the black arrows specifying some of Gram negative bacteria stain red color and blue arrow specifying some of blue/purple stain Gram positive bacterial cells in canals. Outlined circular images with blue and black colors are higher magnification of specified areas coming from colored dotted circles corresponds to the identifications of Gram positive and negative bacteria.

Histological examination of samples revealed that RvD2 induced root apex closure and reduced inflammatory cell accumulation in periapical tissues (Figures 4A,E). The control groups SO and VO did not exhibit root apex closure, and the periapical lesion contained inflammatory granulation tissue with marked inflammatory cell infiltration and numerous PMNs, lymphocytes, and monocytes (Figures 4B,C,F,G). Baseline data (0 week) shows development of periapical periodontitis (Figures 4D,H). Modified Brown and Brenn staining revealed few residual bacteria in RvD2-treated canals compared to the abundant bacteria in control root canals (Figures 4I–L).

### Immunohistochemical Detection of GPR18 and DMP1 in Root Canal Tissues

**Figure 5:**
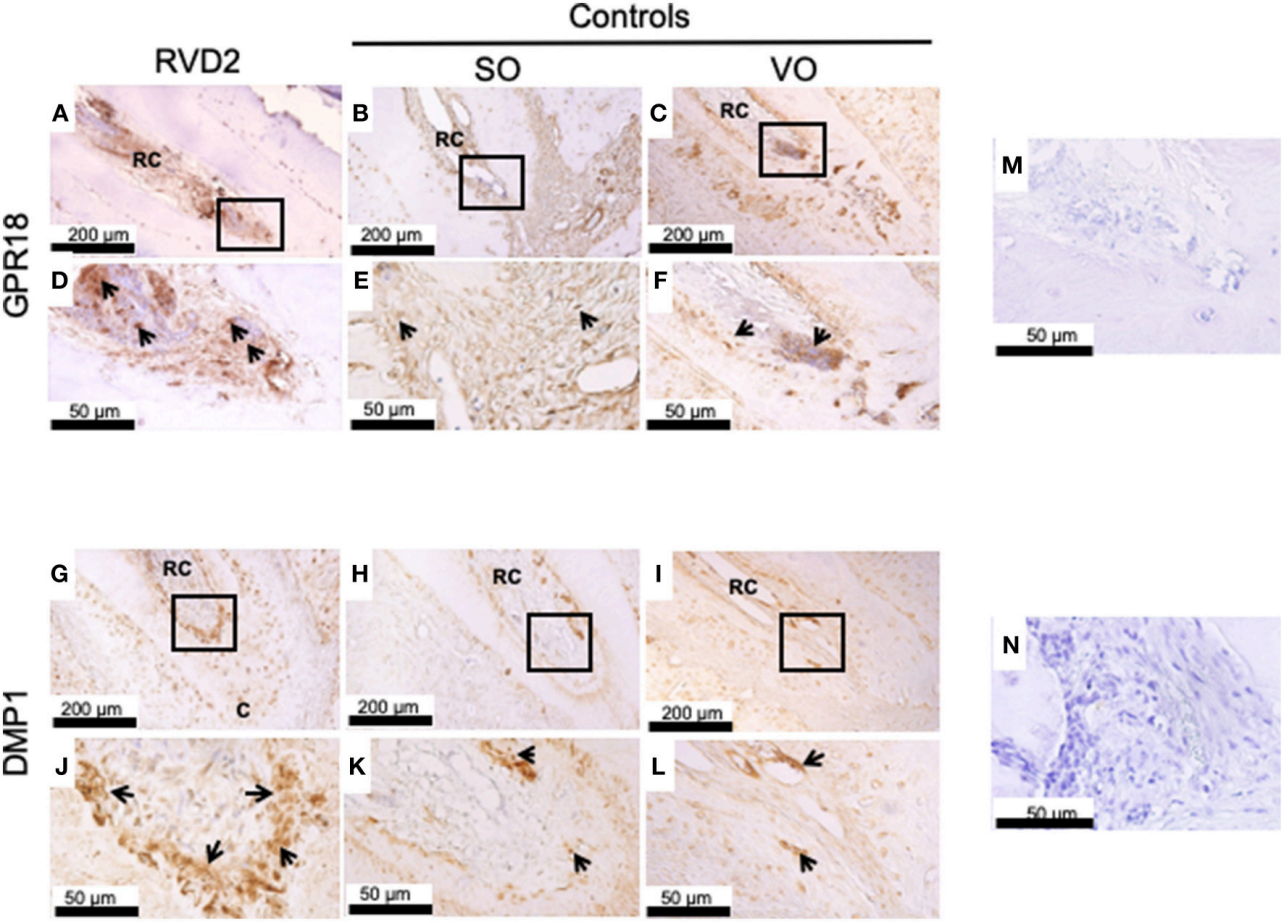
Immunohistochemical analysis. **(A)** GPR18 protein expression in the root canal of the treatment group. **(B,C)** GPR18 protein expression in the root canal of the control groups. **(D–F)** High magnification views of the solid inset in the panels **(A–C)**, respectively. **(G)** DMP1 protein was abundantly expressed in the root canals of the treatment group. **(H,I)** Whereas, DMP1 protein expression was lower in the root canal of the control groups as compared to RvD2 group. **(J–L)** High magnification views of the solid inset in the panels **(G–I)**, respectively. Images are representative for 5 experiments from Group #1 and 3 experiments from Group #2. **(M)** Negative control without primary antibody GPR18. **(N)** Negative control without primary antibody DMP1. RC, root canal; C, cementum. The arrow head indicates GPR18 and DMP1 positive expression.

Immunohistochemistry revealed strong GPR18 protein expression inside and around the root canals in the RvD2-treated group compared to the controls SO, and VO, suggesting the upregulation of the receptor by RvD2 treatment. Expressions were observed inside root canals and in the periodontal ligament space region (Figures 5A–F). DMP1, a key phosphoprotein for dentin mineralization and odontoblast differentiation, was highly expressed in RvD2-treated root canals scattered near the root dentin and mid-root and in the apical region (Figures 5G,J). In the control groups, SO and VO, DMP1 protein was expressed only in the root dentin (Figures 5H–L). Negative controls, without GPR18 and DMP1 antibodies (Figures 5M,N).

### Alizarin Red Staining

**Figure 6:**
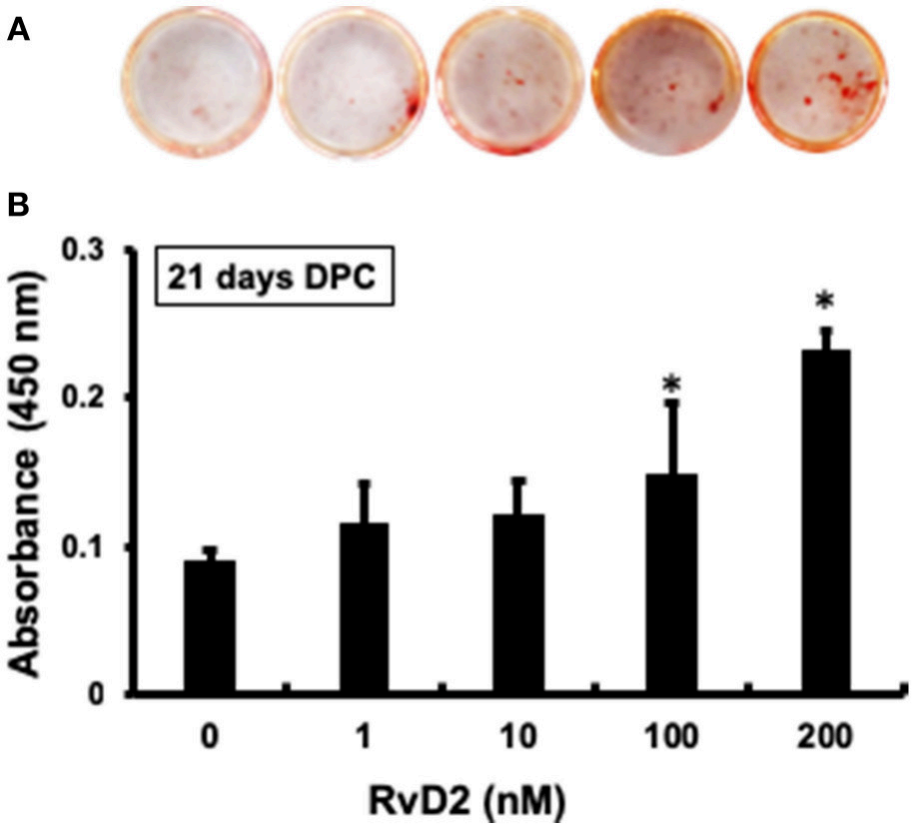
Alizarin red staining of DPCs stimulated with and without RvD2 (1-200 nM) for 21 days. **(A)** Obvious differences in the amounts of mineralization among the groups. **(B)** Quantitative analysis showed that 100 and 200 nM had significantly increased mineralized nodules as compared with control 0 nM (*P* < 0.05; Tukey-Kramer). *P < 0.05 indicates significant differences compared to the control, *post hoc* Tukey-Kramer test. Representative data of four independent samples.

After stimulation of DPCs with RvD2 in culture at doses of 1–200 nM for 21 days, there were obvious differences in the amounts of mineralization among the groups (Figure 6A). Among treatment groups, 100 nM and 200 nM had significantly increased amount of mineralized nodules as compared with control (0 nM) (Figure 6B). Whereas, 1 and 10 nM doses showed slight tendency to induce mineralization in DPCs.

### Expression of DMP1 mRNA in DPCs

**Figure 7:**
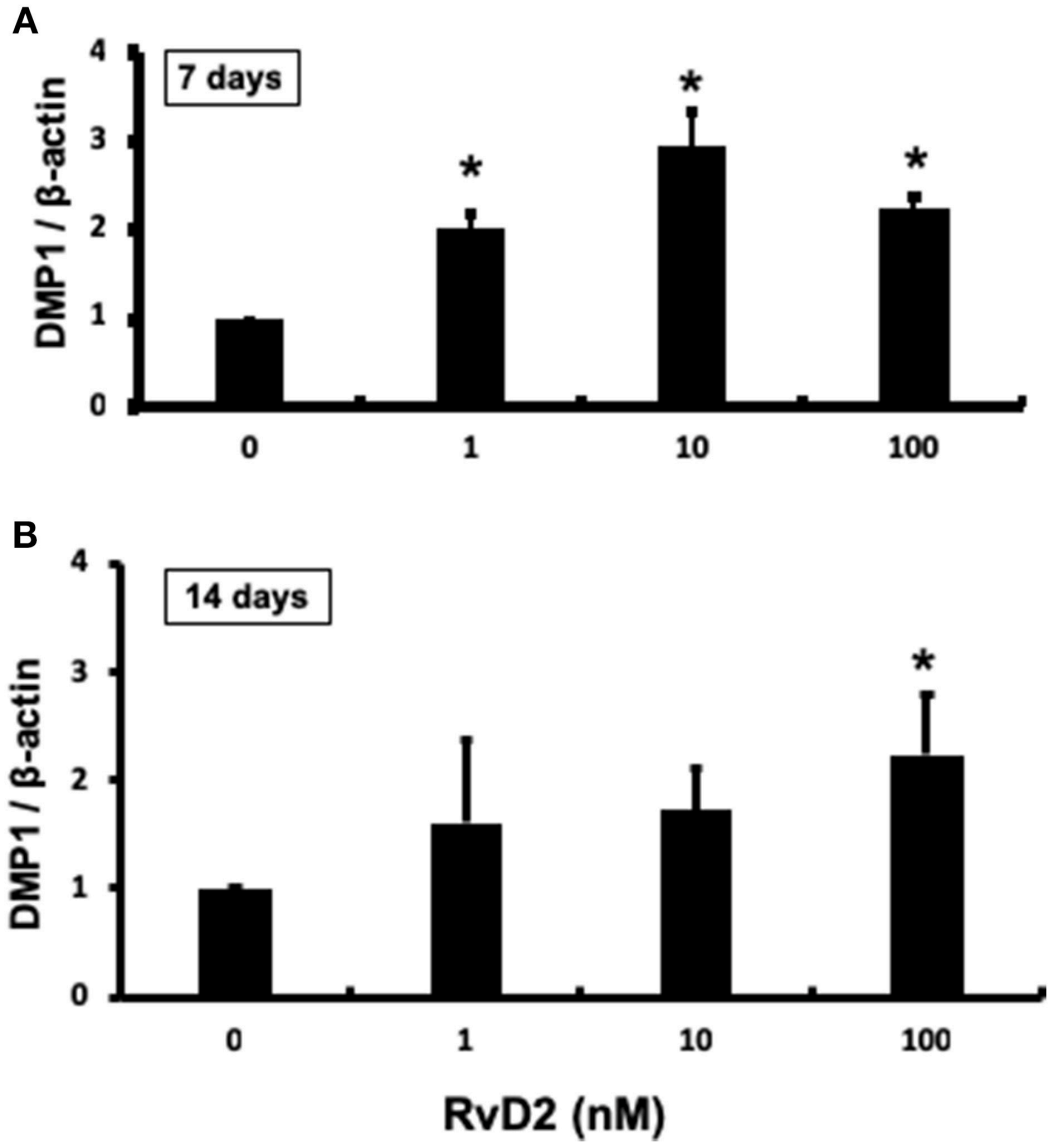
Quantification of DMP-1 mRNA from DPCs stimulated with RvD2 (1–100 nM) for 7 and 14 days using real-time RT-PCR. **(A)** RvD2 induced significant DMP1 mRNA expression at all doses after 7 days culture compared to the control (0 nM) (*P* < 0.05; Tukey-Kramer). **(B)** RvD2 induced significant DMP1 mRNA expression at 100 nM (*P* < 0.05; Tukey-Kramer) after 14 days culture compared to the control (0 nM). *P < 0.05 indicates significant differences compared to the control, *post hoc* Tukey-Kramer test. Representative data of three to four independent cases.

After stimulation of DPCs with RvD2 in culture at doses of 1–100 nM for 7 and 14 days, quantitative real-time PCR analysis revealed that DMP1 mRNA expression was significantly enhanced at each dose at 7 days compared to that in the control (*P* < 0.05, Figure 7A). After 14 days of culture, DMP1 mRNA was significantly enhanced at 100 nM compared to that in the control (*P* < 0.05, Figure 7B).

### Expression of DMP1 and Phosphorylated Signal Transducer and Activator of Transcription 3 (STAT3) Protein in DPCs

**Figure 8:**
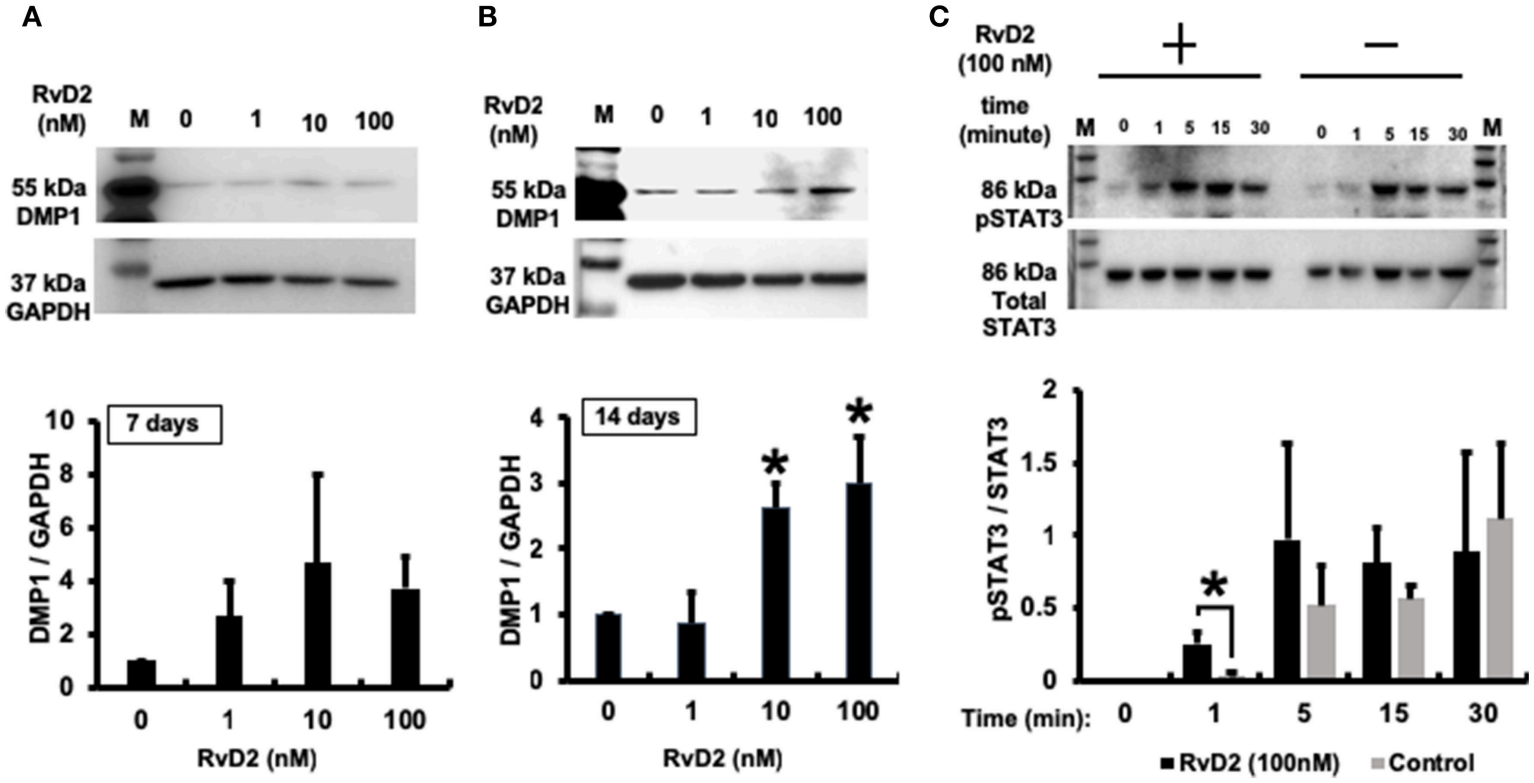
Western blot analysis. **(A)** RvD2-induced DMP1 protein expression in 7 days DPC culture at all doses of 1 to 100 nM. **(B)**. RvD2-induced DMP1 protein expression in 14 days DPC culture. DMP1 expression was significantly enhanced at 10 and 100 nM doses compared to at 1 and 0 nM as the control groups (*P* < 0.05; Tukey-Kramer). Representative data of three independent cases. **(C)** RvD2 induced phosphorylation of STAT3 in DPCs. Cells were stimulated with RvD2 (100 nM) for 0, 1, 5, 15, and 30min. Western blotting revealed that RvD2 significantly induced phosphorylation of STAT3 after 1-min stimulation compared to non-treated DPCs. In addition, phosphorylation of STAT3 was much higher than in non-treated DPCs after stimulation for 5 and 15 min (*P < 0.05 indicates significant differences compared to the control group, Mann–Whitney test). Representative data of three independent experiments. M, molecular weight marker.

Western blotting data confirmed that RvD2 induced DMP1 protein expression in DPCs stimulated with RvD2 for 7 and 14 days. After 7 days, RvD2 induced DMP1 protein expression at all doses of 1 to 100 nM (Figure 8A). Whereas, after 14 days DMP1 protein expression was significantly enhanced at the doses of 10 and 100 nM (*P* < 0.05, Figure 8B). Phosphorylated STAT3 protein expression was significantly induced after DPCs were stimulated with 100 nM of RvD2 for 1 min compared to that in the control. Additionally, phosphorylation of STAT3 was notably higher than that in non-treated DPCs after stimulation for 5 and 15 min (*P* < 0.05, Figure 8C).

## Discussion

In this study, we demonstrate that active control of excess inflammation in an infected root canal is permissive for the healing of periapical lesions. In addition, there was suggestive of successful vital pulp-like tissue regeneration and bacterial load reduction in contaminated root canals following topical RvD2 treatment. Vital pulp-like tissue was regenerated with significant increases in DMP1 expression and mineralization. RvD2 signals, at least in part, through STAT3. The net outcome of RvD2-augmented root canal therapy was continued calcification around root apex, prevention and reversal of periapical periodontitis.

Resolvins are specialized pro-resolving mediators derived from the polyunsaturated omega-3 fatty acids, EPA and DHA, which yield E-Series resolvins and D series resolvins, respectively (30). Resolvins actively foster catabasis through potent pro-resolving and anti-inflammatory actions (31, 32). Specialized pro-resolving mediators derived from DHA, including RvD2, were first noticed and isolated during the resolution phase of self-limited acute inflammation from murine self-resolving exudates (33). Resolvin D2 biosynthesis involves 17-lipoxygenation of DHA to 17S-hydroperoxy-DHA, which then further transformed enzymatically to a 7(8) epoxide-containing intermediate in leukocytes via 5-lipoxygenase, followed by enzymatic hydrolysis to form RvD2. Endogenous RvD2 production has been documented in human serum, plasma, adipose tissue, placenta, lung, breast milk, and in the plasma of sepsis patients. RvD2 is a potent immunoresolvent that stereoselectively reduces excessive PMN trafficking in peritonitis and improves survival in sepsis. It sharply decreased excessive cytokine production, neutrophil recruitment, both local and systemic bacterial burden, while increasing peritoneal mononuclear cells, macrophage phagocytosis and intracellular generation of phagosomal reactive oxygen species for microbial killing in the mice suffered from microbial sepsis initiated by cecal ligation and puncture (16). Moreover, RvD2 promote resolution by preventing the generation of activated Th1 and Th17 cells and enhancing the differentiation of regulatory T-cells (19). In addition, resolvins are protective against *P. gingivalis* induced periodontal bone loss and reverses periodontal bone loss by enhancing bacterial clearance and regulating the RANKL/OPG ratio in murine periodontitis model (17, 34). Overall, RvD2 is an effective endogenous controller of excessive inflammatory responses that actions on multiple cellular targets to stimulate resolution, preserve and improve immune vigilance (16, 35). Importantly, in many disease systems, it has been shown that resolvins can be administered in an active infectious/inflammatory lesion without negative effects. Bacteria are more efficiently cleared and there is no increase of disease activity. The collective data indicate that for all RvD2 actions, such as pro-resolution, anti-inflammation, and the ability to promote bacterial clearance (16), RvD2 appears to be suitable as an intracanal medication for endodontic treatment.

Periapical periodontitis is characterized by inflammation and destruction of periapical tissues caused by etiological bacteria of endodontic origin. It is considered to be the consequence of a dynamic encounter between root canal microbes and host defense. The latter involves cells, specifically PMNs and macrophages, intercellular mediators, metabolites, effector molecules, and humoral antibodies (36). Principally, macrophages play key role in clearance of bacteria, cellular debris and apoptotic PMNs to facilitate inflammation-resolution (16). If dying cells are not cleared, their intracellular contents are expelled, creating an unfavorable environment that may be favorable for bacteria to grow (37). In current clinical practice, there are many materials that do not actively stimulate an immune response such as *gutta-percha* used inside root canals which has little bioactivity and few innate anti-inflammatory properties. These materials lack active anti-inflammatory and regenerative properties and significantly limits treatment options favorable to reverse periapical periodontitis and to drive pulpal regeneration (38).

Intracanal treatment with RvD2 reduced overall inflammation by decreasing MPO activity of phagocytes as compared to control (Figures 2A,B). We believe this change in MPO activity is in accordance with the normal innate immune response. Myeloperoxidase (MPO) activity has been used as an inflammatory marker of both acute and chronic conditions. In PMNs, amount of MPO is 3 times higher than in monocytes, and used as an indicator of PMN presence in inflamed tissues (39). In this study, we visualized *in vivo* monitoring of low MPO activity in RvD2 treated tooth as compared to non-treated tooth. RvD2 limits excessive neutrophil trafficking to site of inflammation and resolve inflammation whereas, unresolved inflammation and tissue destruction are linked to dysregulated PMN functions (16). Further, RvD2 reduced inflammatory cell infiltrates that could be seen histologically (Figures 4A,E). Moreover, RvD2 induced root apex closure and remarkably reduced periapical lesion size with recalcification of bone at 4 weeks after root canal treatment compared to the control group, where large periapical lesions were observed (Figure 3; Figure S2).

In the present study, maintaining root apex patency was considered an important step of the regeneration process, as it has been previously reported by others that the periapical tissues contain a higher concentration of stem cells compared with the blood from the systemic circulation (40, 41). Thus, we instrumented beyond the apex inducing bleeding inside canals. Formation of blood clot creates a 3-D fibrin scaffold that may contain stem cells derived from peripheral blood, periodontal ligament, bone marrow, granulation tissue, or periapical lesions (42). Further, it is likely that the presence of RvD2 restricts excessive innate inflammatory responses in periapical lesions to stimulate periapical repair (16, 17, 19).

Ten-week-old rats were used in this study, because their roots are completely developed at this age. In older rats, it is difficult to use files inside root canals due to continued calcification that makes canals rigid and narrow (1). We measured the periapical lesion size from the apical third of the root canal because of the presence of accessory canals in this area (43). In rats, induced periapical lesions develop rapidly between days 0 and 15 (active phase) and more slowly thereafter (chronic phase). On days 15 to 90, lymphocytes are the predominant cell type (50 to 60%) followed by polymorphonuclear leukocytes (25 to 40%), macrophage-monocytes, plasma cells, and blasts. The cell infiltrate in these lesions was similar to that found in chronic human periapical tissues (44).

Our histological findings are consistent with those of previous studies, which used Resolvin E1 (RvE1) as an intracanal medicament in necrotic immature pulps of teeth of 4-week-old rats and demonstrated reductions in inflammatory cell infiltration (22). Both resolvin types (E1 and D2) bind to distinct cell receptors and elicit distinct responses. RvE1 binds to chemokine-like receptor 1 (ChemR23) or BLT1 receptors on monocyte, PMNs, osteoblasts, and osteoclasts (45). In addition to the anti-inflammatory and pro-resolution actions mediated by RvE1, osteoblasts mediate bone preservation via ChemR23 receptors (45). RvD2 binds to the GPR18 receptor, which is expressed on human leukocytes, including PMNs, monocytes, and macrophages, and elicits potent anti-inflammatory and anti-bacterial responses (16, 35). In this study, we found high expression of GPR18 receptors inside and outside of RvD2-treated root canals as compared to the controls (Figures 5A–F), which was further confirmed by the increased level of GPR18 mRNA expressed by DPCs treated with 10 nM RvD2 for 3 days (Figure S1). These findings are consistent with previous reports that demonstrate stem cells and macrophages express lipoxin and resolvin receptors and respond to exogenous addition of mediators (46, 47). The data expand our understanding of root canal biology beyond the possible anti-inflammatory actions of RvD2 on PMNs and macrophages located inside and outside the root canal, revealing possible reasons for root apex calcification.

For regenerative endodontic therapy, the most commonly used intracanal medication for canal disinfection and tissue repair is triple antibiotic paste, which contains metronidazole, ciprofloxacin, and minocycline (48). Local antibiotic medication has many limitations, including the development of bacterial resistance (49), allergic reactions (50), inhibition of angiogenesis (51), and staining or discoloration of teeth (52). We used 2.5% sodium hypochlorite for root canal irrigation, which has been shown to have an essential role in reducing microbial load (48). It has been studied that lipopolysaccharide (LPS/endotoxin) from bacterial cell walls stimulates an inflammatory response from a variety of cells resident in the tissue (53). Once immune cells become activated by LPS, they mediate the destruction of the pulpal tissues by secreting a range of pro-inflammatory cytokines, prime examples being IL-1β and TNFα, and tissue degrading enzymes such as matrix metalloproteinases (MMPs) (54). Treatment with RvD2 may have further contributed to the control of bacterial infection in the root canal by enhancing phagocyte-dependent bacterial clearance (35). Very low levels of residual bacteria were found in RvD2-treated root canals as compared to control and baseline groups (Figures 4I–L). Gram-negative bacteria predominate in root canals of teeth with pulp necrosis and periapical lesions. LPS is considered an important virulence factor of Gram negative bacteria (55). Moreover, after 4 weeks of root canal treatment, we quantified bacterial cells derived from the mesial root along with apical region, using real-time PCR, as described by Maeda et al. (56). We observed that bacterial cell counts were low in RvD2 treated teeth as compared to control (SO) (Figures S3, S4).

There are many published case reports and series related to the deposition of hard tissue within the canal walls of teeth with necrotic pulps and apical lesions, elucidating the obvious continuation of root development of teeth (57, 58). Other multiple studies in experimental animal models have shown the regeneration of dental pulp-like tissue after evoked bleeding (10, 11), considering cells source from outside the tooth root apex, likely including alveolar bone stem/progenitor cells and periodontal ligament stem/progenitor cells (59). Consequently, vital pulp-like tissue inside RvD2 treated canals and calcified tissues around the root apex (Figures 4A, 5G) presumably derived from alveolar bone stem/progenitor cells and periodontal ligament stem/progenitor cells (60). Sustained vitality of migrated pulp-like cells is likely because RvD2 treatment limits the excess local inflammatory response, controls bacterial sepsis, stimulates stem cells and induces angiogenesis (16, 18). Disease severity and chronicity involve a constant phase of inflammation due to improper resolution of the initial pro-inflammatory response that impacts on the resident stem cells behavior (61), thus preventing tissue regeneration while promoting pathogenesis of periapical lesion caused by excessive influx of PMNs and pro-inflammatory mediators (6, 62). RvD2 actions, could promote resolution of inflammation and reverse tissue destruction caused by excessive PMN influx (16), consequently, facilitate stem cells activations presumably alveolar bone stem/progenitor cells and periodontal ligament stem/progenitor cells (46), and induce regeneration of pulp-like cells and promote calcification of periapical lesion. In this rat model, there was some slight ingrowth of pulp tissue in the control, which is not often seen in humans. Nonetheless, the robust ingrowth and large difference between RvD2 treatment and control demonstrate significant impact of control of inflammation in elimination of infection and promotion of pulp regeneration.

In addition to the known actions of RvD2, it also induced strong DMP1 expression throughout the root canal and around the root apex including in cementum and bone. Moreover, *in vitro* mineralization in primary DPCs was enhanced with RvD2 treatments at 100 and 200 nM doses at 21 days. (Figure 6). 21-day DPC culture was chosen, since osteogenic differentiation and mineralization is optimum at this time (63). We further confirmed that DMP1 mRNA and protein levels were increased in primary DPCs after treatment with RvD2 for 7 and 14 days (Figure 7, 8; Figures S5A,B). DMP1 mRNA expression was significantly enhanced after 7 days treatment with RvD2 at all doses as compared to control (no RvD2). Whereas, at 14 days, the 100 nM dose showed a significant increase in DMP1 expression. The difference in dose/response time could be due to high cellular confluency in culture dishes incubated for longer periods. It could be suggested that for the induction of mineralization, persistent DMP1 expressions are needed. This time 10 to 100 nM dose induced DMP1 protein expression at 14 days (Figure 8B). We hypothesize that high concentration of RvD2 is needed to keep DMP1 production and induce periapical calcification. DMP1 is an extracellular acidic phosphoprotein that belongs to the small integrin ligand N-linked glycoprotein family 1, which has multiple functions in mineralized tissues. It is also expressed in the cementum that coats the tooth root surface (64). It plays a key role in odontoblast differentiation and formation of the dentin tubular system. In response to pulp injury, newly differentiated odontoblast-like cells from DPCs play a role in dentin repair and mineralization by secreting DMP1 as a key protein that induces odontogenesis. Overexpression of DMP1 by pluripotent cells acts as signal for differentiation (14). In this case, differentiated odontoblast-like cells may have shifted from the end of dentinal tubules toward the root apex as dentin formation progressed (Figures 5G,J). Narrowing of the root canal space was observed in some RvD2-treated molars, likely in response to increased DMP1 protein. DMP1 is highly acidic in nature and attracts calcium and promotes the nucleation and growth of hydroxyapatite crystals (65). Localization of DMP1 in dentin and cementum is related to mineralization and its deletion leads to increased susceptibility to periodontal diseases in mice, suggesting that DMP1 is essential for the formation and maintenance of a healthy periodontium (66, 67).

Finally, we identified a signaling pathway in DPCs involved in the induction of DMP1 over-expression and odontoblast differentiation. We observed that phosphorylation of STAT3 was enhanced after DPCs were treated with RvD2 (Figure 8C; Figure S5C). The differences in STAT3 phosphorylation were small; however, in normal cells, the duration of STAT3 activation is short. Usually after exposure, phosphorylation takes peaks within minutes (68). In our study, we found that DPCs expressed phosphorylated STAT3 protein 1 min after exposure. While expression was also higher after 5 and 15 min, we assume that RvD2 activates STAT3 signaling in DPCs as early as in 1 min after exposure, and may persist until RvD2-receptor interactions are saturated. STAT3 stimulates embryonic and somatic stem cell self-renewal (69, 70). STAT3 also activates DPCs and promotes their exit from the G0 phase of the cell cycle toward self-renewal and differentiation (71). RvD2-GPR18 receptor interactions on macrophage leads to the phosphorylation of STAT3 that contributes in macrophage phagocytosis to promote resolution of inflammation (35). Hence, in DPCs, RvD2-GPR18 receptor interactions phosphorylates STAT3 to further propagate DPC differentiation into odontoblast-like cells. STAT3 is a positive regulator of β-catenin (72, 73), and initiates DPC differentiation and upregulates DMP1 expression. Further studies are needed to explore the signaling pathways responsible for DPCs differentiation, DMP1 upregulation and the mechanisms involving mineralization.

In conclusion, RvD2 efficiently reduces periapical inflammation and promotes pulp-like tissue regeneration and calcification around root apex. The positive healing response could be associated with reduction of bacteria load. RvD2 enhances DMP1 expression by DPCs. RvD2 may be useful as a novel intracanal medication for inducing pulpal regeneration in endodontically compromised teeth.

## Ethics Statement

This study was approved by the Animal Care and Use Committees, Okayama University (Permit no: OKU-2017062). All animal experiments were carried out in accordance with the Guidelines for Animal Experiments of Okayama University, surgical procedures were performed under sodium pentobarbital anesthesia, and all efforts were made to minimize animal suffering.

## Author Contributions

YS contributed to the conception, design, analysis, and interpretation of the study and wrote the manuscript. KaO, TI, and KY contributed to the conception, design, analysis, and interpretation of the study and drafted the manuscript. SN and KeO contributed to *in vivo* experiments. MO contributed to data analysis. TY contributed to interpretation of the study. TV contributed to the conception, interpretation of the study, and drafted and critically revised the manuscript. ST contributed to the conception, design, analysis, and interpretation of the study and drafted and critically revised the manuscript.

### Conflict of Interest Statement

The authors declare that the research was conducted in the absence of any commercial or financial relationships that could be construed as a potential conflict of interest.
