# Supplementary material for: Resolvin D2 Induces Resolution of Periapical Inflammation and Promotes Healing of Periapical Lesions in Rat Periapical Periodontitis
Source: Front Immunol. 2019 Feb 26;10:307. doi: 10.3389/fimmu.2019.00307 (PMC6399419; doi:10.3389/fimmu.2019.00307)
Supplement: Supplementary file 1 [file Presentation_1.pptx]

## Slide 1
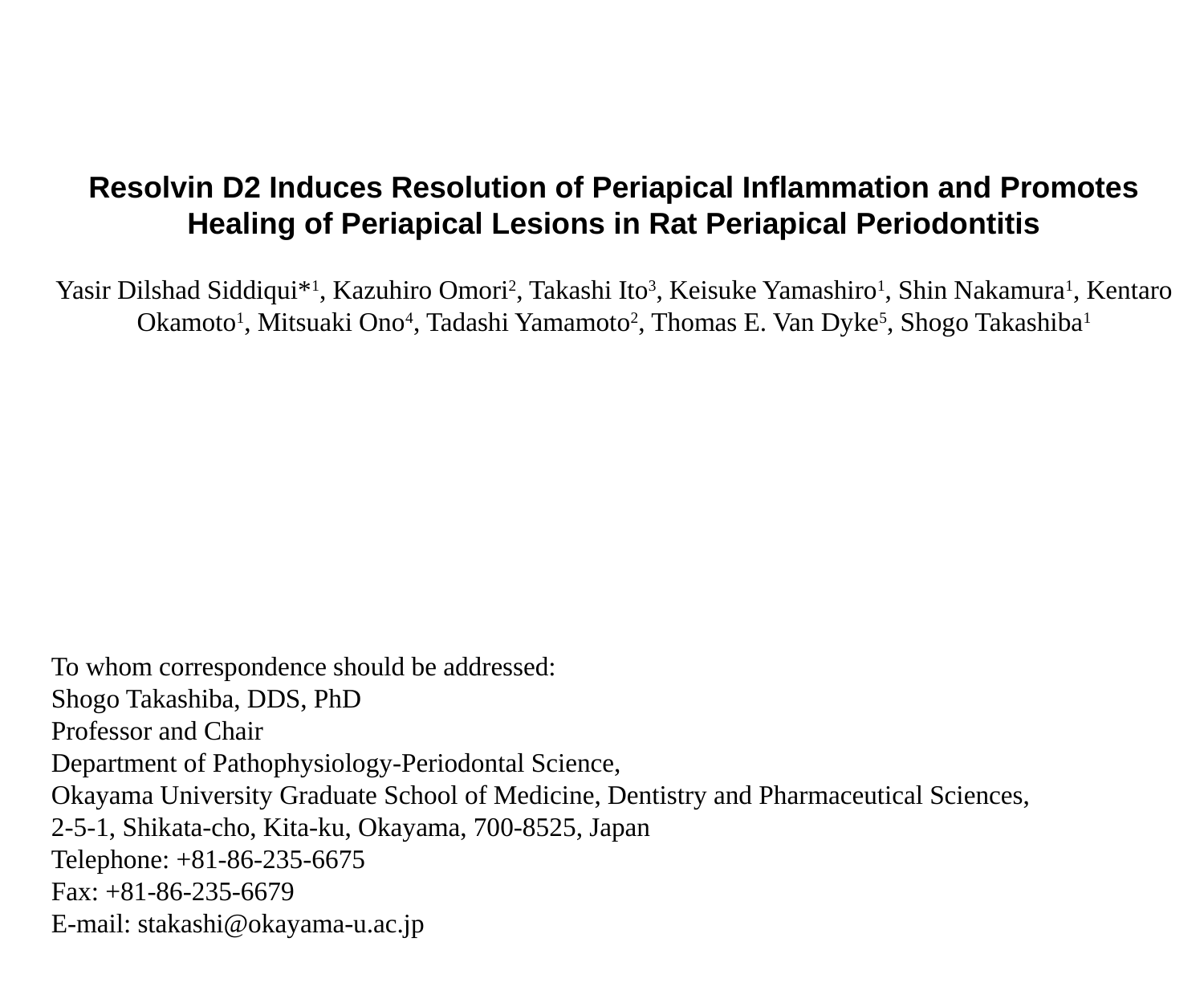

Resolvin D2 Induces Resolution of Periapical Inflammation and Promotes Healing of Periapical Lesions in Rat Periapical Periodontitis
Yasir Dilshad Siddiqui*1, Kazuhiro Omori2, Takashi Ito3, Keisuke Yamashiro1, Shin Nakamura1, Kentaro Okamoto1, Mitsuaki Ono4, Tadashi Yamamoto2, Thomas E. Van Dyke5, Shogo Takashiba1
To whom correspondence should be addressed:
Shogo Takashiba, DDS, PhD
Professor and Chair
Department of Pathophysiology-Periodontal Science,
Okayama University Graduate School of Medicine, Dentistry and Pharmaceutical Sciences,
2-5-1, Shikata-cho, Kita-ku, Okayama, 700-8525, Japan
Telephone: +81-86-235-6675
Fax: +81-86-235-6679
E-mail: stakashi@okayama-u.ac.jp

## Slide 2
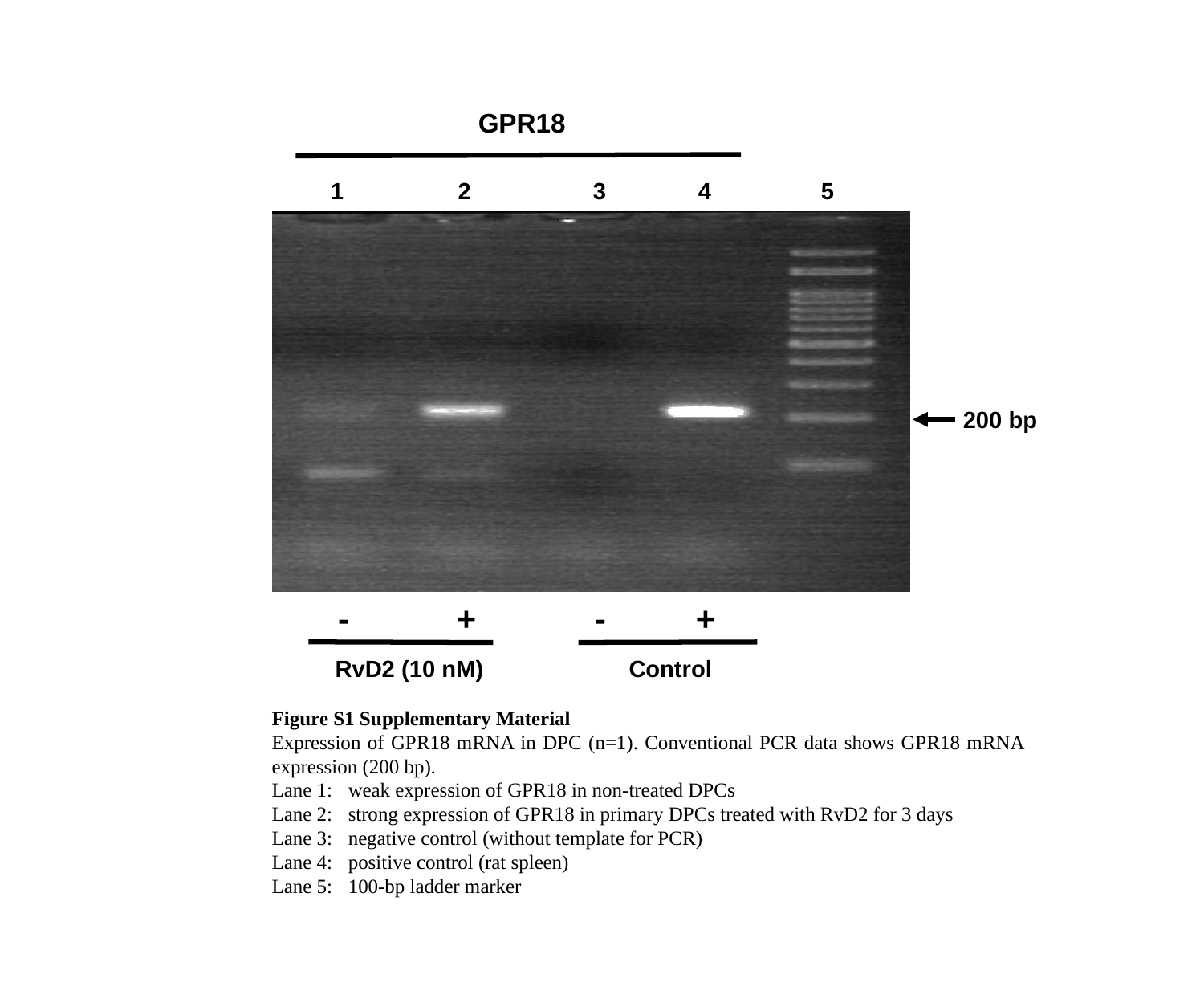

GPR18
1	2	3	4	5
200 bp
-	+	-	+
RvD2 (10 nM)
Control
Figure S1 Supplementary Material
Expression of GPR18 mRNA in DPC (n=1). Conventional PCR data shows GPR18 mRNA expression (200 bp).
Lane 1:	weak expression of GPR18 in non-treated DPCs
Lane 2:	strong expression of GPR18 in primary DPCs treated with RvD2 for 3 days
Lane 3:	negative control (without template for PCR)
Lane 4:	positive control (rat spleen)
Lane 5:	100-bp ladder marker

## Slide 3
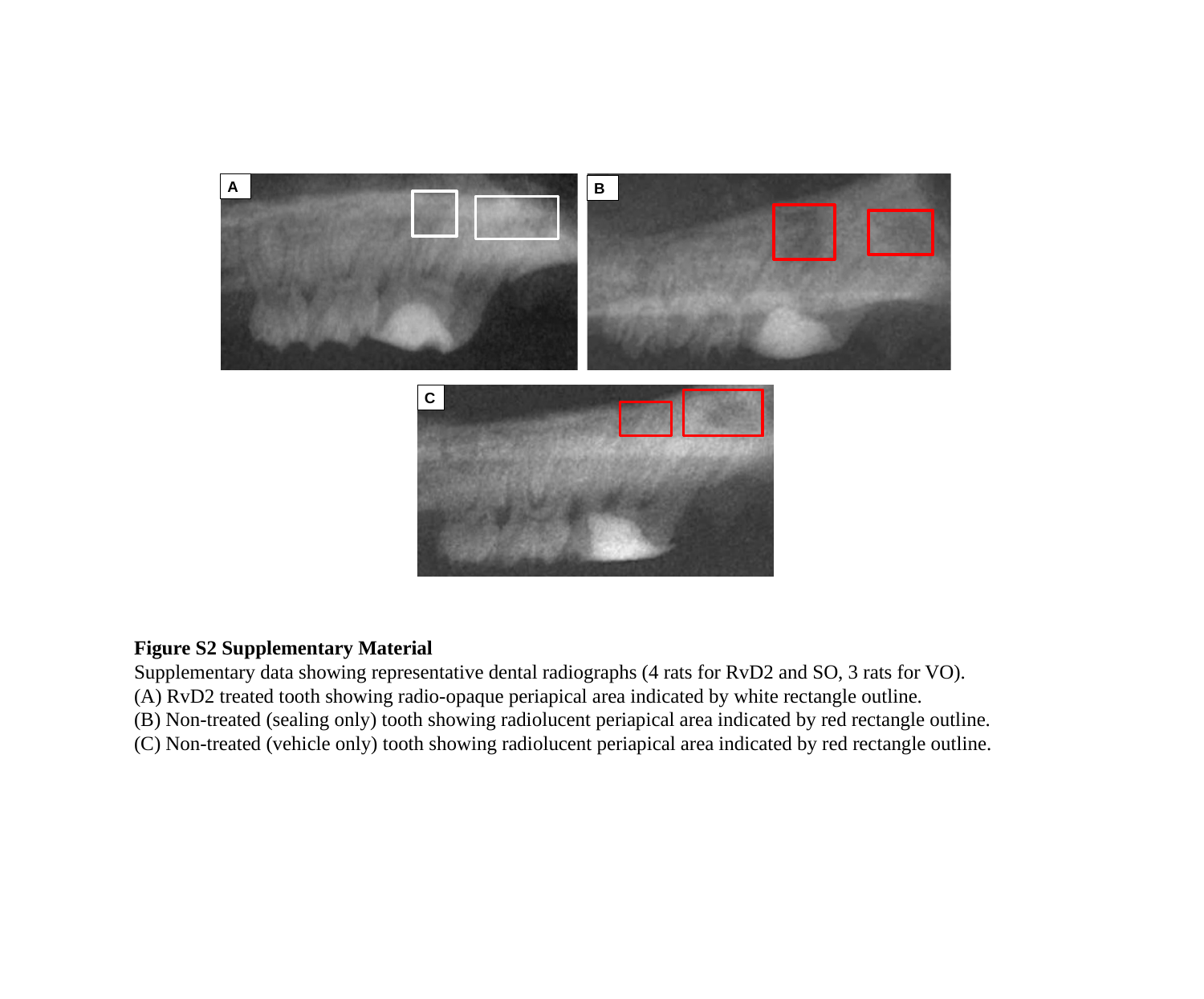

A
B
C
Figure S2 Supplementary Material
Supplementary data showing representative dental radiographs (4 rats for RvD2 and SO, 3 rats for VO).
(A) RvD2 treated tooth showing radio-opaque periapical area indicated by white rectangle outline.
(B) Non-treated (sealing only) tooth showing radiolucent periapical area indicated by red rectangle outline.
(C) Non-treated (vehicle only) tooth showing radiolucent periapical area indicated by red rectangle outline.

## Slide 4
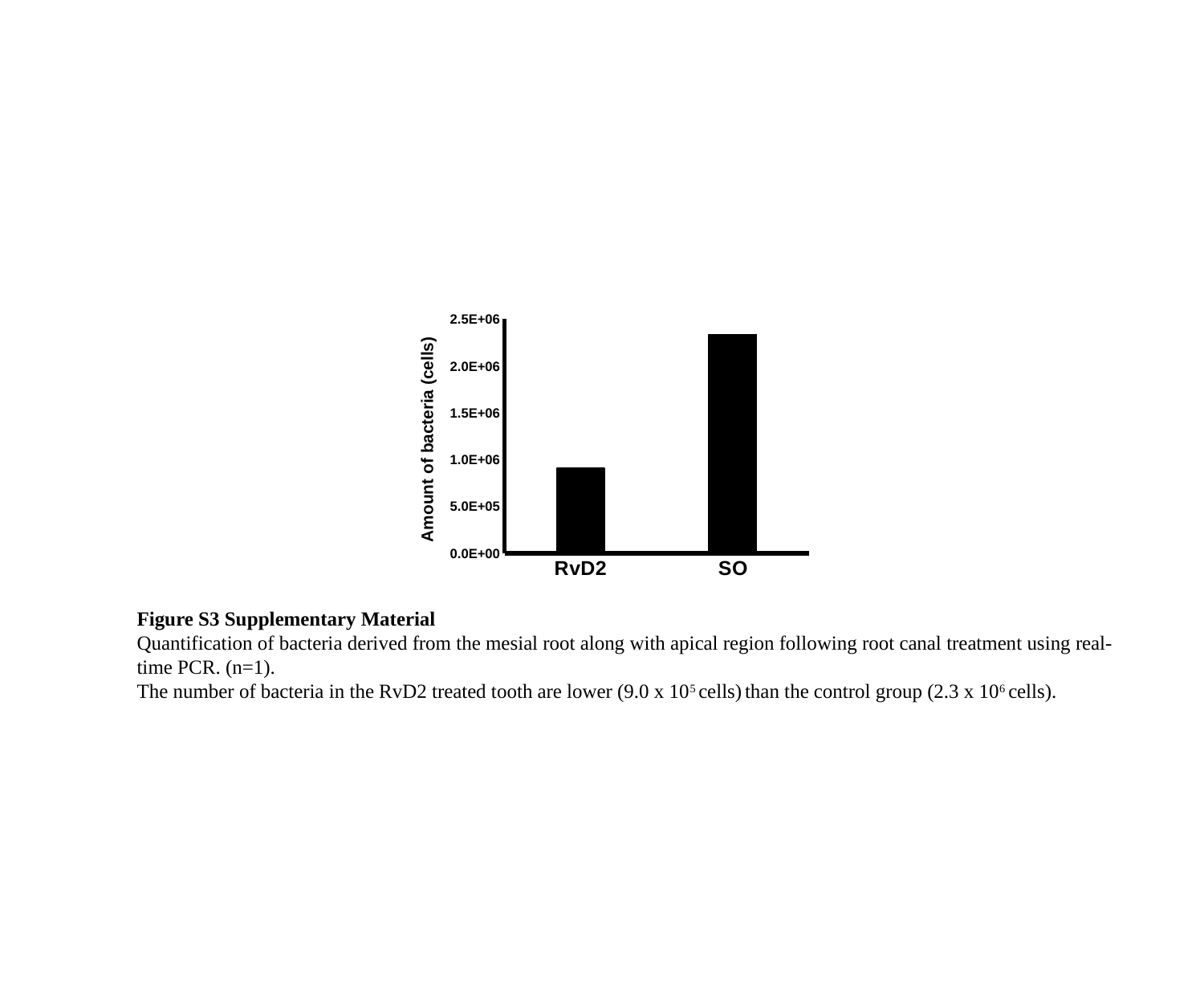

### Chart
| Category | |
|---|---|
| RvD2 | 906656.1799099846 |
| SO | 2338477.23803354 |Amount of bacteria (cells)
Figure S3 Supplementary Material
Quantification of bacteria derived from the mesial root along with apical region following root canal treatment using real-time PCR. (n=1).
The number of bacteria in the RvD2 treated tooth are lower (9.0 x 10^5^ cells) than the control group (2.3 x 10^6^ cells).

## Slide 5
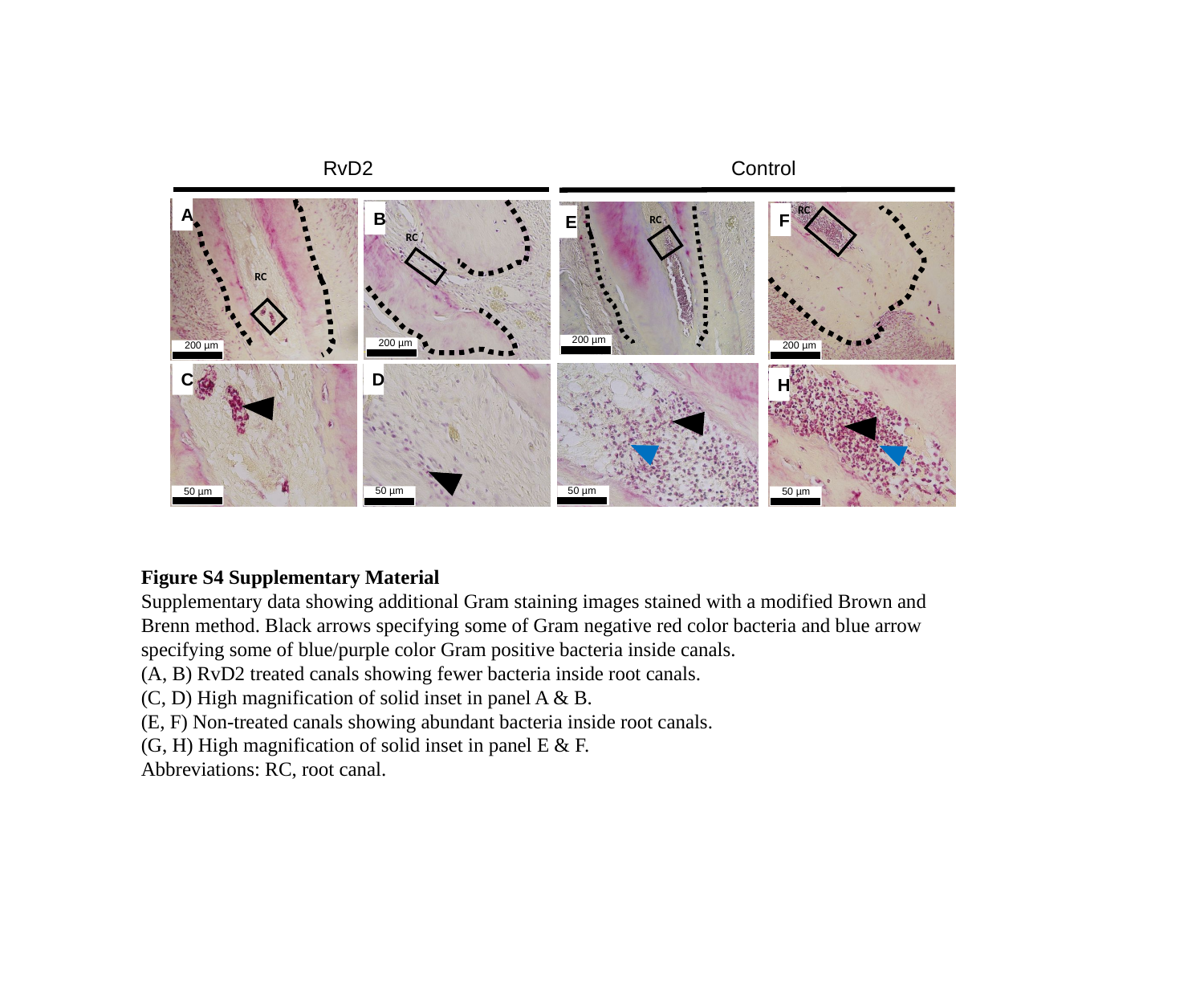

Control
RvD2
A
RC
B
F
E
RC
RC
RC
200 µm
200 µm
200 µm
200 µm
C
D
G
H
50 µm
50 µm
50 µm
50 µm
50 µm
Figure S4 Supplementary Material
Supplementary data showing additional Gram staining images stained with a modified Brown and Brenn method. Black arrows specifying some of Gram negative red color bacteria and blue arrow specifying some of blue/purple color Gram positive bacteria inside canals.
(A, B) RvD2 treated canals showing fewer bacteria inside root canals.
(C, D) High magnification of solid inset in panel A & B.
(E, F) Non-treated canals showing abundant bacteria inside root canals.
(G, H) High magnification of solid inset in panel E & F.
Abbreviations: RC, root canal.

## Slide 6
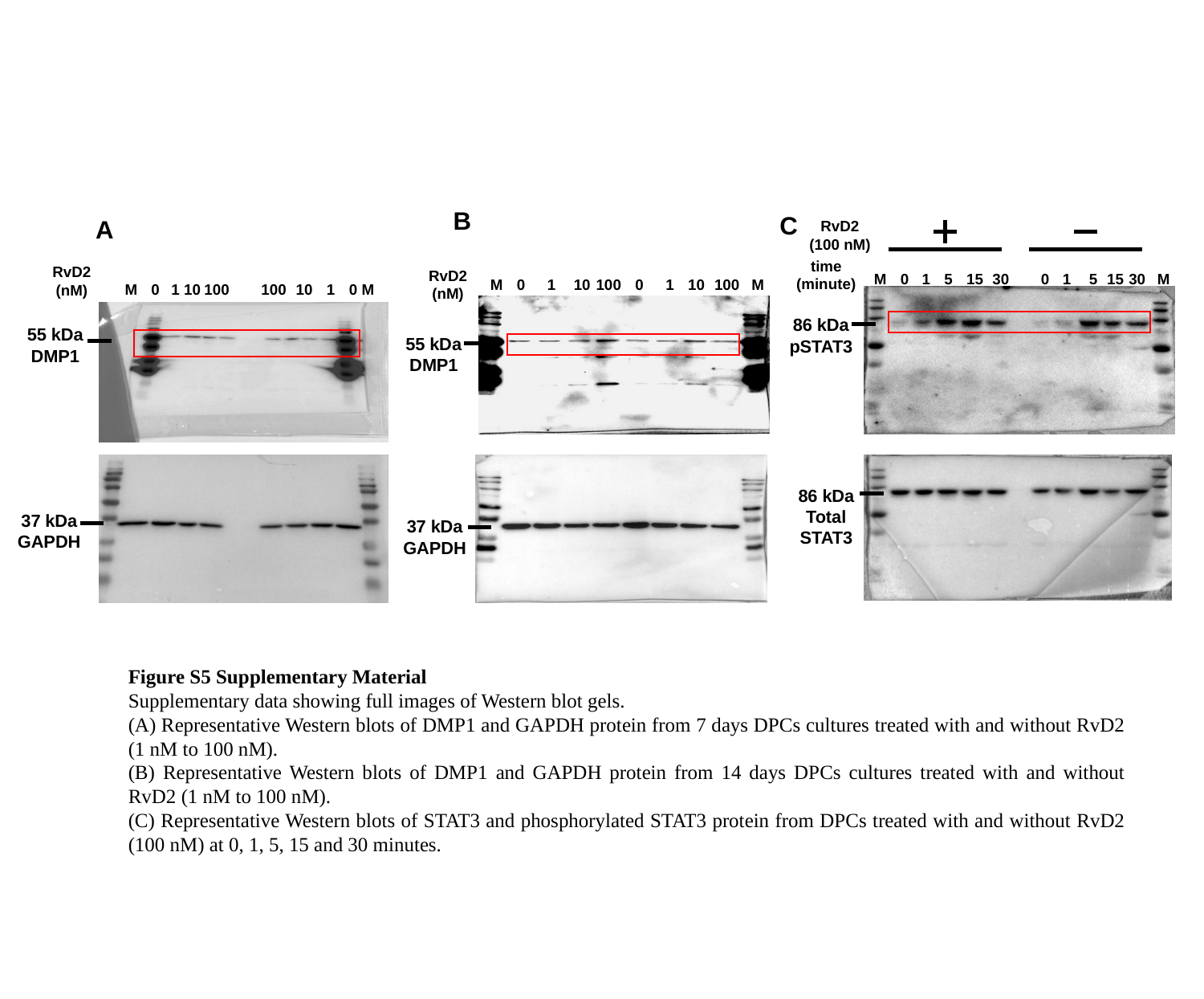

B
C
A
RvD2
(100 nM)
time
(minute)
M	0	1	5	15	30	0	1	5	15	30	M
RvD2 (nM)
RvD2 (nM)
M	0	1	10	100	0	1	10	100	M
M	0	1 10	100		100	10	1	0 M
86 kDa
pSTAT3
55 kDa
DMP1
55 kDa
DMP1
86 kDa
Total STAT3
37 kDa
GAPDH
37 kDa
GAPDH
Figure S5 Supplementary Material
Supplementary data showing full images of Western blot gels.
(A) Representative Western blots of DMP1 and GAPDH protein from 7 days DPCs cultures treated with and without RvD2 (1 nM to 100 nM).
(B) Representative Western blots of DMP1 and GAPDH protein from 14 days DPCs cultures treated with and without RvD2 (1 nM to 100 nM).
(C) Representative Western blots of STAT3 and phosphorylated STAT3 protein from DPCs treated with and without RvD2 (100 nM) at 0, 1, 5, 15 and 30 minutes.
